# The Need for Hematology Nurse Education in Low- and Middle-Income Countries: A Community Case Study in Tanzania

**DOI:** 10.3389/fpubh.2017.00065

**Published:** 2017-03-29

**Authors:** Julie M. Buser

**Affiliations:** ^1^Department of Health Behavior and Biological Sciences, University of Michigan School of Nursing, Ann Arbor, MI, USA

**Keywords:** hematology nursing education, hemato-oncology nursing education, hematology nurses, low-income countries, middle-income countries, Tanzania

## Abstract

Hematology-related diseases, such as anemia, malaria, sickle cell disease (SCD), and blood cancers, have differing rates of survival between high-income and low- and middle-income countries (LMICs). Nurses in LMICs have an unmet need for specialty training and education to address hematology and hemato-oncology disorders. A gap in the literature exists about hematology nurse education and clinical service demands in LMICs. This community case study documents a collaborative hematology and basic hemato-oncology education program to sustainably strengthen nurse capacity at a national referral hospital and university in Tanzania. The goal of the intervention was to provide culturally competent nurse training in pediatric and adult hematology. A certified pediatric nurse practitioner with hematology and oncology experience provided culturally competent training and staff development to nurses over two weeks to meet this goal. Prior to development of a training schedule, nurses confidentially identified five of their top learning needs. Main hematology and basic oncology educational needs identified by nurses were the management of anemia, safe handling of cytotoxic agents, and treatment of SCD. The format of the education varied from bedside teaching to formal presentations to one-on-one individual discussions. Overall, nurses expressed satisfaction with the education and verbalized appreciation for teaching and training activities tailored to meet their needs. Specialized training in hematology and hemato-oncology has the potential to increase nurses’ confidence, respect, and participation in interprofessional team decision-making. Lessons learned from the impact of collaborative nurse education and partnership in Tanzania can be generalized to other LMICs. This community case study highlights the importance of specialty nurse education, interprofessional development, and global partnerships needed to improve patient outcomes.

## Introduction

Nurses play an important role in sustainably improving patient outcomes in low- and middle-income countries (LMICs). Nurse education in LMIC tends to focus on primary care topics. However, there is a growing need to manage chronic disease and reduce the burden of hematology-related diseases globally and improve public health. Nurses in LMICs have an unmet need for specialty training and education to address hematology and hemato-oncology disorders. Nurses with adequate levels of specialty hematology nursing education will better meet the health-care needs of patients. The purpose of this community case study is to document a collaborative hematology and basic hemato-oncology education program to sustainably strengthen nurse capacity at a national referral hospital in Tanzania.

## Background and Rationale

Hematology-related diseases such as anemia, malaria, sickle cell disease (SCD), and blood cancers have differing rates of survival between high-income and LMICs (1). Globally, anemia affects 1.62 billion people, which corresponds to 24.8% of the population (2). The highest prevalence is in preschool-age children (47.4%), and the group with the greatest number of individuals affected is non-pregnant women (468.4 million) (2). SCD is one of the most common diseases worldwide yet in many LMICs, basic facilities for management are lacking, systematic screening is not common practice, and diagnosis is made late (3). Leukemia and lymphomas occur in LMICs but it is difficult to estimate the burden of disease. However, we know lower-income families endure a double burden of communicable and non-communicable chronic illness, requiring a response well integrated into the health systems of LMICs (4). The practice of hematology is evolving in many countries in Africa to encompass the growing and changing demands in clinical and laboratory services as well as blood transfusion (5).

Globally, nurses comprise the largest group of health-care providers. In almost all countries, nursing and midwifery services are estimated to comprise over 80% of the health-care services (6). About 43% of World Health Organization (WHO) Member States report to have less than two nursing and midwifery personnel per 1,000 population (28% report to have less than 1) (6). The average nurse:population ratio in high-income countries is almost eight times greater than in low-income countries (7). In Tanzania, the density of nursing and midwifery personnel (total number per 1,000 population) in 2012 (last available year) was 0.436 (6). The low availability of nurses in many developing countries is exacerbated by geographical misdistribution—there are even fewer nurses available in rural and remote areas (7).

The strain of the nursing shortage on health-care systems is a worldwide concern. The need for specialty nurses is often overlooked in the face of this public health challenge. There is a gap in the literature about clinical service demands in relation to nursing and hematology in LMICs. It has yet to be demonstrated how nursing will evolve to meet public health demands. The lack of specialty training for nurses is being overlooked by the largest group of health-care providers. There are challenges associated with inadequately prepared nurses needed to care for the population suffering from hematology-related problems. Education and interprofessional collaboration should be used to meet the evolving service demands of specialty nursing in LMICs.

### Description of the Case

The goal of the educational intervention was to provide hematology and basic hemato-oncology training and staff development to nurses with Muhimbili National Hospital (MNH) and nursing students at Muhimbili University of Health and Allied Sciences (MUHAS) in the largest city in Tanzania, Dar es Salaam. The nurse educator observed inpatient pediatric and adult nurses had little training in how to care for patients with hematological and hemato-oncological disorders and recognized a definite need for improved nursing care. The hematology and basic hemato-oncology training and staff development for nurses was developed based on the self-identified needs of the nurses and by the nurse educator through observation of patient care.

### Setting

MUHAS (8) aims to provide quality training, research, and services in health and related fields for attainment of equitable socioeconomic development for the Tanzanian community and beyond. The vision of the MUHAS School of Nursing (8) is to become a center of excellence in nursing education, research, consultancy, and public services responding to national, regional, and global challenges. MNH is a national referral hospital, research center, and university teaching hospital with a 1,500 bed facility, attending 1,000–1,200 outpatients per day, and admitting 1,000–1,200 inpatients per week (9). As the national referral center, MNH is responsible for providing specialized care for hematological disorders. The hospital operates inpatient wards for both children and adults with hematological conditions. Children under the age of eight years are treated in the pediatric division which consists of a sickle cell ward and a pediatric oncology ward. The hematology unit runs three outpatient clinics and also provides care to patients in obstetrics and gynecology, surgery, orthopedics and trauma, and otolaryngology (10).

## Collaborative Partnerships

The organization fostering inclusive partnerships in this community case study is Health Volunteers Overseas (HVO). They are a United States-based non-profit organization dedicated to improving the availability and quality of health care through the education, training, and professional development of the health workforce in resource-scarce countries (11). At HVO, nursing education is central to their work to improve the availability of nursing care around the world. Their projects plan for the next generation of nursing staff, providing essential information and skills to future nurse educators (HVO nurse partners). The aim is to create a sustainable solution to global nurse shortages, giving local health systems the capacity to teach and train the staff they need (11).

The American Society of Hematology (ASH) partners with HVO to bring consultation and training to hospitals in the developing world managing hematology patient cohorts with a wide range of disorders. Training takes the form of rounds in the clinics, bedside consultations, lectures in the classrooms, training in the laboratories, and more. The objective of the sponsorship is sustainable improvement of the management of hematology patients in the developing world (12).

### Methodological Aspects

The format of the education at MNH varied from bedside teaching to formal presentations to one-on-one individual discussions. Flexibility was key to meeting the scheduling demands of approximately 50 Tanzanian nurses caring for pediatric and adult hematology patients in both in- and outpatient settings. Group lectures were presented more than once to reach a wide audience. At MUHAS, lectures were presented to both undergraduate and postgraduate nursing students.

## HVO Nurse Partner

Education sessions were delivered by a certified pediatric nurse practitioner with over 10 years’ experience working in the fields of hematology and oncology along with clinical instruction at a large teaching hospital in the United States. The HVO nurse partner also has extensive experience working in LMICs with a major international medical humanitarian organization. The courses were delivered in English because nursing school, as with most tertiary education in Tanzania, is taught in English. At the national hospital, the HVO nurse partner participated in hematology department grand rounds, pediatric and adult hematology clinical rounds, outpatient pediatric and adult sickle cell clinics, as well as the new patient screening clinic and anti-coagulation clinic. Table 1 shows some highlights of the HVO nurse partner’s schedule while in Tanzania.

**Table 1:** **Highlights of the Health Volunteers Overseas nurse partner’s schedule in Tanzania**.

Day 1	Day 3	Day 7	Day 9	Final day
Meeting with nurse managers, head nurses of peds/adult hematology/oncology wards	Observe nurses in adult sickle cell follow-up clinic	Group lecture with nurses about safe handling of chemotherapy	Observe hematology grand rounds	Informal qualitative evaluations of educational program with nurses
Discussions with pediatric oncology nurses to review their learning objectives	Introduction to faculty at Muhimbili University of Health and Allied Sciences (MUHAS). Collaborative identification of teaching topics with students	Individual instruction with adult hematology nurses	Participate in pediatric hematology inpatient rounds with staff MDs and inpatient nurses	Review of experience with assistant director of volunteer program
Tour of all wards in pediatric hospital with hematology nurse	Visit clinical pathology/hematology lab offices	Observe nurses in new patient screening clinic	Attend hematology dept. MD research presentations	Lecture at MUHAS

## Collaborative Approach

Prior to the development of a training schedule, Tanzanian nurses were asked to confidentially prioritized five of their top learning needs related to hematology and basic oncology. Results of the prioritization survey were incorporated into the training schedule. The main educational needs identified by nurses were the management of anemia, safe handling of cytotoxic agents, and treatment of SCD. Specific goals of the nurse education program reached using the prioritization process are detailed in Table 2. Group lectures were held daily and lasted between 0.5–1 h and were repeated two to three times throughout the program so that as many nurses as possible could attend lectures. Groups averaged nine participants. The smallest was 5 and largest 21. The HVO nurse partner collaborated with Tanzanian nurses to arrange timing of group and individual education sessions.

**Table 2:** **Highlights of hematology nurse education program schedule in Tanzania**.

Day 2	Day 4	Day 6	Day 8	Final day
Nurses self-identified education needs using anonymous survey	Cross-cultural discussions with individual nurses about differences in resource management between Tanzania and US	Group lecture: management of treatment side-effects, 0.5 h session, presented two times during program	Individual instruction with pediatric oncology nurses about safe chemotherapy administration	Informal qualitative interview evaluations of educational program with nurses
Cross-cultural discussions with individual nurses about differences in treatment options between Tanzania and US	Group lecture: overview of hematologic cancers, 1 h session, presented three times during program	Individual sessions at bedside with nurses to review sickle cell crisis pain management	Small group discussions about impact of cross-cultural hematology education program	Group lecture: management of nurse self-care/coping strategies, 45 min session, presented once during program
Group lecture: overview of hematology, 1 h session, presented three times during program	Individual sessions at bedside with nurses to review management of treatment side-effects	Lecture at Muhimbili University of Health and Allied Sciences (MUHAS) to 56 undergraduate third year nursing students about emergency management	Group lecture: anemia treatment, 0.5 h session, presented two times during program	Lecture at MUHAS to nine post-graduate second year students about transfusion medicine

## Educational Program

A 2-week collaborative educational program was developed to provide hematology and basic hemato-oncology education to nurses in Tanzania. The education program included three main themes: (a) overview of hematologic and hemato-oncologic disorders; (b) safe chemotherapy administration; and (c) supportive care. The education sessions included four components: (1) basic hematology and hemato-oncology group lectures; (2) individual instruction on self-identified needs; (3) direct observation of patient care in pediatric and adult inpatient wards and hematology outpatient clinics; and (4) group lectures to graduate and postgraduate nursing students. Table 3 gives highlights of the hematology nurse education program schedule in Tanzania.

**Table 3:** **Specific goals of hematology nurse education and professional development**.

Nurse identified educational needs	Specific goals of educational sessions
Sickle cell disease	Review nursing care management of sickle cell, pain crisis and infection management, coping skills
Transfusion medicine	Increase understanding and knowledge of transfusion reactions, safe administration of blood
Hemato-oncology	Foster improved understanding of nursing care of patients with blood cancer
Chemotherapy	Provide details on safe administration and care, exchange transfusion, and home care
Bleeding and thrombosis	Monitor and detect signs of bleeding, clot formation
Anemia and other red cell disorders	Review nursing care implications and overview of pathophysiology for blood disorders

At MUHAS School of Nursing, at the request of nursing faculty, 56 undergraduate students received one 2-h presentation including an overview of hematologic disorders, blood product administration, and how to recognize blood transfusion reactions. Another 2-h presentation was given to nine graduate nursing students on the recognition and treatment of hematologic and basic oncologic emergencies such as neutropenic fever and unexplained bleeding.

## Educational Topics

A summary of educational topics covered at MNH and MUHAS is presented in Table 4. For example, one topic identified by Tanzanian nurses as an area they wanted to learn more about were the principles of chemotherapy and safe handling of cytotoxic agents. The HVO nurse partner observed nurses in the chemotherapy preparation area and noted areas for improvement. The HVO nurse partner suggested ways to decrease surface contamination and encouraged the use of eye protection when preparing chemotherapy. After observation of nurses in the oncology ward, the educator proposed ways to reduce the potential for chemotherapy extravasation including the insertion of fresh IV catheters prior to vesicant administration and monitoring IV sites for brisk blood return and good flow during infusion.

**Table 4:** **Nurse education at Muhimbili National Hospital and Muhimbili University of Health and Allied Sciences**.

Topic	Content covered in lectures and individual education sessions with nurses and nursing students
**Overview of hematology**	
Origin of blood cells	PowerPoint presentation from American Society of Hematology Lecture Program: Blood Basics
Anemia	The role of red blood cells in anemia, common types of anemia, treatment for anemia, prevention of nutritional anemias
Neutropenia	Causes of neutropenia, infection prevention
Thrombocytopenia	Physical examination, symptom review
**Hematologic disorders**	
Sickle cell disease (SCD)	Overview of SCD and sickle cell trait, patterns of inheritance, risk factors, signs and symptoms, treatment, hydroxyurea
Thalassemia	Causes of thalassemia, patterns of inheritance
Hemophilia	Overview of bleeding disorders and hemophilia, patterns of inheritance, activity precautions
Emergency management	Treatment of acute bleeding events, sickle cell pain crisis, acute chest syndrome
**Overview of hematologic cancers**	
Leukemias, lymphomas	Overview of cancer cells, immune system impairment
Hematopoiesis and immune response	Creation of new blood cells, blood formation, response to antigens
Principles of chemotherapy	How chemotherapy works, medical treatment, dosage differences for pediatric and adult patients
Safe handling of cytotoxic agents	Safety considerations in preparation, administration, and disposal of chemotherapy
**Supportive care**	
Blood product components	Overview of red cells, white cells, platelets, and plasma
Blood product administration	Best practices for transfusion, transfusion reactions
Nutritional support	Health dietary options, importance of nutrition in healing process
Management of treatment side-effects	Treatment of nausea, vomiting, diarrhea, infection, fatigue, mucositis, neuropathy

## Evaluation

At the end of the hematology nurse education program, participants were asked in an informal survey to confidentially write the three things they liked most about the education sessions. They were also asked to verbally evaluate their experience during informal small group discussions on the final day of training. Nurses were asked to describe the potential outcomes of the educational program and to comment on any potential nursing practice outcomes after collaborating in the educational program. No quantitative data were collected as part of the evaluation.

## Theoretical Framework

The US-based HVO nurse partner used Leininger’s (13) transcultural nursing movement in education research and practice as a framework for providing culturally competent hematology nurse education. Transcultural nursing focuses on comparative human-care (caring) differences and similarities of the beliefs, values, and patterned lifeways of cultures to provide culturally congruent, meaningful, and beneficial health care to people (13). Leininger’s (13) Culture Care Diversity and Universality Theory served as a guide for the selection of intervention elements keeping in mind every human culture has folk remedies, professional knowledge, and varying professional care practices. Using Leininger’s model in Tanzania, the HVO nurse partner identified and addressed these factors consciously with each nurse to provide holistic and culturally congruent education.

## Teaching Framework

The teaching framework used by the HVO nurse partner followed the approach outlined in “Educating Health Professionals in Low-Resource Countries: A Global Approach” (14). Three common principles of adult learning guided delivery of the intervention by the HVO nurse partner including (1) learners must be engaged by understanding the value and benefits of their learning; (2) outcomes and goals are clearly defined; and (3) teachers should use evidence-based teaching principles to facilitate learning (14). Active listening, observation, and culturally congruent communication were used in the bedside and classroom presentations. Individual and group training sessions fostered mutual respect, cultural competence, and exchange of ideas between the teacher and Tanzanian nurses and nursing students.

## Discussion

This community case study documents the collaborative hematology and basic hemato-oncology education intervention to sustainably strengthen nurse capacity at a national referral hospital in Tanzania. Overall, using informal qualitative surveys, nurses in the hematology and hemato-oncology areas expressed satisfaction with the education after the intervention was complete. The nurses ranked the following as what they liked most about the program: (1) opportunity for continuing education; (2) cross-cultural discussions; and (3) group lectures. The informal verbal feedback from participants following the educational intervention was extremely positive. Nurses verbalized appreciation that teaching and training activities were tailored to meet their needs. Some of the comments reflecting the nurses’ evaluation of the training included:

I am so glad you came to talk to us. Nobody ever pays attention to us nurses. Only the doctors get educated over and over again.

I learned a lot about giving chemotherapy that I didn’t know before. Now, I am not so scared to give treatment as I used be.

The presentations were very helpful.

Thanks for listening to our concerns and teaching about what’s important for us to know.

Students in the School of Nursing at MUHAS also reported satisfaction with the presentations given by the HVO nurse partner. After class, students were each asked to write three of their top take-home messages from the education intervention. Narrative comments from the nursing students included:

It’s so nice to listen to international perspectives about nursing. We like to hear from experts in different subject areas. The best was about how to make sure blood types are compatible.

I will remember to talk to my patients about getting good nutrition for healing if they have cancer of the blood.

My best take home message is that sickle cell disease can stay in one family and hydroxyurea is a good medicine for those patients to take.

Specialized training in hematology and hemato-oncology has the potential to increase nurses’ confidence, respect, and participation in interprofessional team decision-making. The WHO and its partners recognize interprofessional collaboration in education and practice as an innovative strategy playing an important role in mitigating the global health workforce crisis (15). Interprofessional education is a necessary step in preparing a “collaborative practice-ready” health workforce better prepared to respond to local health needs (15).

While there is little information regarding hematology nurse education programs in LMICs, there are a couple of programs reporting results of oncology nurse education. Recognizing oncology nurses in LMICs have limited access to specialized education and clinical training, the International Outreach Nursing Program at St. Jude Children’s Research Hospital created the Latin American Center for Pediatric Oncology Nursing Education in Santiago, Chile to provide education, resources, and support to local nurse educators (16). In turn, these nurse educators educate the entire nursing staff at partner sites throughout Latin America. The educators provided pediatric oncology education to more than 1,000 nurses who can improve the quality of care and ultimately survival of patients throughout Latin America (16).

Recognizing there is no existing pediatric oncology nursing curriculum written specifically for LMICs, the International Society of Pediatric Oncology (SIOP) Pediatric Oncology in Developing Countries (PODC) conducted a survey of LMIC nurses in 2012 (17). A cross-sectional sample of LMIC nurses from Africa, Latin America, and Asia indicated a similar need for specialty training and curriculum development (17). The importance of education and learning is underscored by the development of an Education and Training Working Group in SIOP PODC which aims to facilitate training and education of health-care providers in LMICs (18). This case study adds to the growing body of evidence showcasing the importance of specialty nurse training in LMICs.

A team of Australian nursing and midwifery educators delivered evidence-based education in Tanzania and found with appropriate levels of cultural competence, international health professionals can be effective at providing ongoing professional development to colleagues in developing countries (19). Researchers suggest that in preparation for an international teaching intervention, implementers should incorporate principles based on the work of Campinha-Bacote (20) when learning how to work in partnership with local health professionals to (1) assess learning needs; (2) understand and acknowledge differences in approach between educators and course participants; and (3) development of practical and relevant course content to suit local conditions (19).

Sustainable development calls for concerted efforts toward building an inclusive, sustainable, and resilient future for people and planet (21). Goal 17 of the recently launched United Nations Member Sustainable Development Goals (SDGs) encompasses revitalizing the global partnership for sustainable development to enhance international support for implementing effective and targeted capacity building in developing countries (21). The United Nations Member States recognize the importance of fostering inclusive partnerships built upon principles and values, a shared vision, and shared goals placing people and the planet at the center, are needed at the global, regional, national, and local level (21).

## Lessons Learned/Recommendations

Urgent action is needed to educate nurses caring for patients with hematologic disorders such as anemia, SCD, blood cancers, and more in Tanzania and other LMICs. Nurses play an important role in achieving optimal care of patients with hematology-related problems yet there are large numbers of patients being cared for by nurses with no training in hematology. The work of HVO, in partnership with ASH, to improve the quality of health care in Tanzania through the education, training, and professional development of nurses in hematology is commendable. There is a definite need to advocate for other educational partnerships to increase the quality and supply of specialized nurses in LMICs.

Quality education is the foundation for developing competent health workers who are equipped with the knowledge, attitudes, and skills necessary to deliver quality care (22). In future intercultural exchanges, trainers should adopt the recently announced WHO Nurse Educator Core Competencies which were developed to help guide the educational preparation of nurse teachers, ensure educational quality and accountability, and, ultimately, contribute to improving the provision of nursing care and outcomes of health services (22).

The interdisciplinary collaboration barrier identified by nurses should not be ignored by team members in Tanzania. A great deal of effort should be focused on building a “collaborative practice-ready” health workplace at Muhimbili and at other nurse education sites. Collaborative practice strengthens health systems and improves health outcomes (15). Collaborative practice happens when multiple health workers from different professional backgrounds work together with patients, families, caregivers, and communities to deliver the highest quality of care. It allows health workers to engage any individual whose skills can help achieve local health goals (15).

The case study was time-limited because teaching strategies and cultural exposure took place over a period of two weeks. A longer period of immersion in the setting and culture would allow time for formal curriculum development and training to benefit nurses providing care to patients with hematology-related problems in LMICs. Follow-up education sessions would allow for the reinforcement of material as would be creating sound or video recordings of sessions. Another limitation is some of the nurses expressed a lack of time to attend educational sessions. In the future, efforts should be made to schedule dedicated time to learn without the responsibility of providing patient care. Of course, this will be a challenge as the nursing workforce far too often experiences high patient to nurse staffing ratios. However, with proper advanced planning, a greater number of nurses should be able to benefit from training initiatives.

A limitation of the community case study is that it is written from only one side of the cultural exchange. Several unsuccessful attempts were made to contact lead nurses in the hematology department at MNH. The case study would no doubt be strengthened with the active input of Tanzanian participants in the writing process.

Future studies should evaluate the effect of hematologic and hemato-oncologic nurse education in LMICs. Recommendations for future research in LMICs would be to examine the effects of educating nurses in hematology and whether there is an associated improvement in patient outcomes. Another recommendation would be to examine the costs associated with educating nurses. Eventual research should investigate the cost-effectiveness of nursing education and impact on patient outcomes. The incorporation of a systematic evaluation process including both qualitative and quantitative measures would add richness and inform future collaborative efforts.

Lessons learned from the community case study experience in Tanzania can be generalized to other LMICs. Nurse educators and HVO nurse partners providing training in other settings must implement their programs with a willingness to provide culturally appropriate interventions in diverse settings. The education of hematology nurses should be scaled up in Tanzania and other LMICs settings. This community case study is the first one to document a hematology education intervention to sustainably strengthen nurse capacity in an LMIC and highlights the importance of specialty nurse education, interprofessional development, and global partnerships needed to improve patient outcomes.

## Author Contributions

JB, the sole author, made substantial contributions to the conception, design, and drafting of the work including final approval of the version to be published.

## Conflict of Interest Statement

This work was conducted in the absence of any commercial or financial relationships that could be construed as a potential conflict of interest.
